# Is empathy an important attribute of midwives and other health professionals?: A review

**DOI:** 10.18332/ejm/100612

**Published:** 2019-02-12

**Authors:** Anastasia Charitou, Polyxeni Fifli, Victoria G. Vivilaki

**Affiliations:** 1 Department of Midwifery, University of West Attica, Athens, Greece

**Keywords:** empathy, midwives, health professionals, midwifery, patient care

## Abstract

**INTRODUCTION:**

This paper is a report of a systematic review to identify and analyze studies of the measurement of empathy in midwives and other health professionals. Empathy has been recognized as an important factor in patient care, with positive outcomes for both patients and health professionals. There is a debate on the definition of empathy, on its measurement and on the possibility of improvement.

**METHODS:**

Searches were made of the CINAHL, SCOPUS, PubMed and PsychINFO databases using the terms empathy, clinical, midwifery, nursing, medical students, measurement, and health professionals, singly or in combination, to identify literature published in English between 2002–2015. The included papers were critically reviewed and a narrative synthesis was conducted.

**RESULTS:**

In all, 22 papers met the inclusion criteria by studies that were conducted to measure the levels of empathy in a variety of health professionals and students. Their scores were analyzed in correlation with their sociodemographic factors.

**CONCLUSIONS:**

Despite numerous studies, many correlations but also differences exist, indicating the complexity of empathy and the need to further study it.

## INTRODUCTION

Empathy is considered a key attribute in health care and can benefit both patients and health professionals. The concept of empathy has its origin in the German word ‘einfühlung’, which means ‘feeling into’, and was established by the German psychologist Theodore Lipps, as a standard term of psychology^1-3^. Later Tichener translated Lipps’s term by coining the word ‘empathy’ from two Greek roots, ‘em’, meaning ‘to put into, to bring about a certain condition or state, to furnish with something’ and ‘pathy’ (from pathos), meaning ‘suffering or passion’^2-8^. Although there is mainly agreement in the literature that empathy has positive effects there is no consensus about its definition^9-11^. It has often been described as ‘elusive and mysterious’^12^ and any attempt to define it is considered ‘an intellectual challenge’^13^.

Carl Rogers^14^ described empathy as an ability ‘to perceive the internal frame of reference of another with accuracy as if one were the other person but without ever losing the ‘as if’ condition^2,15-20^. Friedmeier commented: ‘Empathy is an affective reaction that stems from the perception of another emotional state or situation of another person, that involves vicariously experiencing another person’s situation and that is characterized by the attention paid to another person’s emotions’3. Kalish^21^ wrote: ‘Empathy is the ability to enter into the life of another person, to accurately perceive his current feelings and their meanings. In empathy, the helper borrows his patients feelings to understand them fully, but he is always aware of his separateness’.

Hojat proposed the following definition of empathy in the context of patient care ‘Empathy is a predominantly cognitive (as opposed to affective) attribute that involves an understanding of experiences, concerns and perspectives of the patient, combined with a capacity to communicate this understanding’. Cognition, understanding and communication are the key components in this definition of empathy that is widely used^13,22-24^.

It is important to distinguish empathy from sympathy in caring for patients because they have different clinical outcomes^25-27^. Sympathy is the act or the capacity of entering into or joining the feelings of another person. Empathy is described as a capacity to understand but without joining the feeling of the patient’s situation^25,28,2,3,10,29^. Empathetic health professionals share their understanding while sympathetic health professionals share their emotions with the patients^30^.

Researchers have studied the neurobiological aspect of empathy and have suggested that empathy may be hard-wired into the human nervous system^31-32^. They have observed that empathy activates the same regions of the brain that process aspects of pain^31^. Compelling new evidence of the brain’s mirror-like neuronal response to observing others in pain, regardless of prior experience cognitive appraisal, suggests that certain aspects of an empathetic response may be autonomic and beyond our control^33^.

Studies have shown that clinical empathy could enhance the therapeutic outcomes of a patient’s relationship with a health professional^22,34-42^ and could also achieve better patient satisfaction^43-45^, better compliance and adherence to treatment^39,9,35,45,40,30^, and facilitates the development of mutual trust^34,46-48^. There are also important implications for pain management practices^34^. Generally, being an empathetic health professional improves patient confidence^3,40^, reduces patient’s anxiety^34^, helps the patient feel respected and validated^10,41^, and willing to provide more specific information about his/her medical history^10,30^. It has also been suggested that there is less malpractice litigation^10,35,49^.

Although many empathy scales have been developed, there are limitations and difficulties in the measurement of empathy because of the ambiguity in its definition^11,50^. Some of the empathy instruments that have been used included Barrett-Lennard Relationship Inventory, Emotional Intelligence Scale, Empathic Tendency Scale, Interpersonal Reactivity Index, LaMonica Empathy Profile, and more recently the Jefferson Scale Of Physician Empathy (JSPE), which is the most frequently used by physicians and medical students as well as by other health professionals. Recently, the first empathy scale was created, specifically designed to measure midwives’ empathy (Midwifery Empathy Scale - MES)^51^.

The ability to offer empathy may vary from one individual to another as some people are by nature more empathetic than others. However, acquired empathy can be taught as a skill and developed with practice and experience^36,20,52-55^.

### Empathy and midwifery care

Empathy plays a key role in midwifery care. In this situation, the healthcare professional does not come in contact with a patient who is experiencing a pathological condition but with a healthy woman who is in need of great support. A pregnant woman needs constant support and care, physically and mentally, before and after pregnancy. The midwife is the health professional who is basically in contact with the woman throughout her pregnancy, her labour, and after the birth. The development of a good relationship with her midwife is essential for a woman’s better birth and perinatal experience and also helps in the management of pain^56-58^. The central points of this relationship are trust, mutuality, support, recognition of the uniqueness of a woman and confirmation^58^. Thus, it is perceived how important it is for a midwife to have all those characteristics that promote good communication with the woman, such as friendliness, tenderness, calmness, readiness, and empathy^59-62^. Empathy is particularly important to midwives and allows them to understand things from a woman’s angle^58^. Midwives with high levels of empathy, ‘stand in women’s shoes’ and can understand how women feel, which is very helpful, especially in some phases of childbirth when women do not wish to speak, or in cases where verbal communication is impossible^58^. The non-verbal expression of empathy by the midwife such as her touch (holding the hand and the caress) during the childbirth improves the woman’s ability to cope with her condition, helps her to feel more comfortable and reduces her blood pressure and pulse rate^63^. Despite the importance of empathy in midwives, it is important to highlight that the number of studies of midwifery empathy is minimal.

## METHODS

Searches were done within the Scopus, PubMed, CINAHL, PsycINFO databases using the terms ‘clinical’, ‘empathy’, ‘nursing’, ‘medical students’, ‘physicians’, ‘midwifery’, by themselves or in combination, to identify the relevant literature. The following inclusion and exclusion criteria were used:

Inclusion criteria: journal articles with quantitative research design; studies applying a scale to measure empathy levels; participants being physicians, medical, nursing, midwifery and emergency-help students; publications in English between 2002–2014.

Exclusion criteria: review articles; studies that included intervention and measurement before and after; studies focusing on empathy, but where no tools were applied to assess its level, as in qualitative studies.

The search identified 12077 papers in total, whose titles and abstracts were read to obtain the relevant studies that were used in the systematic review. Additional searches conducted were based on the references of the selected works.

## RESULTS

The research found a significant increase in the score of empathy skills in last year medical students and a non-significant difference between male and female medical students, despite indicating that female students have higher empathy scores. The increase in empathetic abilities and decrease in active conflict tendencies in years four and six was remarkable, suggesting that clinical education comprising patient–physician relationships may be helpful in a period that is more empathetic and less conflictually active^64^ (Table 1).

**Table 1:** List of studies that measure the levels of empathy

***Atay et al. (2014)^64^***
Aim To investigate the empathy levels and conflict tendencies in medical students considering the phase of medical education.
Setting and Country Medical School, Turkey
Design and Sample 186 medical students
Rating Methods Empathic Skills Scale-B form, ESS-B & Conflict Tendency Skills,CTS
Results No significant differences of empathy scores between male and female students. The mean score of empathy for 6th year students was 125.6±26.3 and was significantly higher than both1st and 4^th^ year scores (t=6.14, p=0.015; t=18.95, p=0.002, respectively). There was a significant negative correlation between the scores of existential conflict and empathy skills.
***Chen et al. (2007)^65^***
Aim To measure and examine student empathy across medical schoolyears.
Setting and Country Boston University School of Medicine, USA
Design and Sample 658 medical students
Rating Methods Jefferson Scale of Physician Empathy - Student, JSPE-S
Results The 1st year medical school class has the highest empathy scores. Female medical students have higher empathy than male (116.5 vs 112.1, p<0.01). Students preferring people-oriented specialties have a higher empathy than those preferring technology-oriented specialties (114.6 vs 111.4, p=0.02)
***Costa et al. (2012)^66^***
Aim Aims to longitudinally model empathy during medical school 3 time points. At the entrance, final of the preclinical phase and at the beginning of clinical training.
Setting and Country University of Minho School of Health Sciences, Portugal.
Design and Sample 77 medical students
Rating Methods Jefferson Scale of Physician Empathy - Student, JSPE-S & Neuroticism-Extraversion-Openness Five-Factor Inventory (NEOFFI)
Results Non-significant differences on empathy scores (t(76)=1.04, p=0.30) were found between the preclinical (M=111.21, SD=10.80) and clinical phases (M=110, SD=10.85). A significant decline in empathy scores from preclinical (M=113.41, SD=10.57) to clinical phase (M=110.77, SD=10.84) was noticed, but only in regard to female students (t(52)=2.17, p=0.035, d=0.25). Students with higher values of openness and agreeableness showed higher values of empathy in start point.
***Costa et al. (2014)^67^***
Aim To assess associations between students’ personality and empathy across institutions, looking for personality differences between students with high empathy and low empathy levels.
Setting and Country 3 Medical Schools, Portugal
Design and Sample 472 medical students
Rating Methods Jefferson Scale of Physician Empathy - students Portuguese version, JSPE-spv & Neuroticism-Extraversion-Openness Five- Factor Inventory (NEO-FFI)
Results For a total of 334 students a significant and positive correlation was found between total JSPE score and Extraversion (r=0.183, p<0.01), Openness to Experience (r=0.216, p<0.01), Agreeableness (r=0.310, p<0.01) and Conscientiousness (r=0.188, p<0.01).
***Di Lillo et al. (2009)^6^***
Aim To examine the psychometrics of the JSPE among a sample of Italian physicians.
Setting and Country 3 hospitals in Rome, Italy
Design and Sample 289 physicians
Rating Methods Jefferson Scale of Physician Empathy, JSPE
Results Women scored higher that men by 3 points (117.5 vs 114.5) but the difference was not statistically significant (t(287)=1.33, p=0.17). A difference of almost 3 points was also detected among medical and surgical specialties (117.5 vs 114.2) but it was not statistically significant (t(286)=1.49, p=0.13). Comparisons of physicians in the 3 hospitals showed marginally significant differences.
***Hasan et al. (2013)^68^***
Aim To evaluate the level of empathy among medical students in Kuwait University Medical School and each association with sociodemographic factors, stress levels and personality.
Setting and Country Kuwait University Medical School, Kuwait
Design and Sample 264 medical students
Rating Methods Jefferson Scale of Physician Empathy - Student, JSPE-S & Zuckerman-Kuhlman Personality Scale, ZKPS & Perceived Stress Scale, PSS
Results There was a significant difference between the male and the female students; the mean score for males was 100.6±18.5 and for females 107.1±14.1 (p≤0.003). The empathy levels were also significantly different between the years of study. The 4th year students scored the highest, while the 2nd year students scored the lowest (p≤0.037). Statistically significant association was found between family income and empathy. Students with household income <KD 100 per month had lower empathy scores than those with higher income (p≤0.005). A statistically significant association was found between empathy and educational level of the mother (p≤0.018). Students who were satisfied with their relationship with their mothers scored higher than those who were neutral or not satisfied (p≤0.005). Stress levels were found to be significantly and positively associated with empathy as students with higher stress levels scored higher on the empathy scale (p≤0.041).
***Hojat et al. (2002a)^22^***
Aim To investigate the components of physician empathy, its measurement properties and group differences in empathy scores.
Setting and Country Thomas Jefferson University Hospital, USA
Design and Sample 704 physicians
Rating Methods Jefferson Scale of Physician Empathy, JSPE
Results The mean empathy score for men (mean=119.1, SD=11.8) was slightly lower than for women (mean=120.9, SD=12.2) and the difference between genders was nearly significant (t=1.71, df=684, p=0.08). Age did not significantly correlate with empathy scores for men (r=0.01) or women (r=0.07) statistically significant differences were found in empathy scores among physicians in different specialties (F=1.99, df=11, 493, p<0.05). Psychiatrists had the highest mean empathy score (mean=127.0). The lowest were scored by physicians in general surgery, and obstetrics and gynecology had scores that fell between these high and low scoring specialties.
***Hojat et al. (2004)^69^***
Aim To examine changes in empathy among medical students as they progress throughout medical school.
Setting and Country USA
Design and Sample 125 medical students (3rd year)
Rating Methods Jefferson Scale of Physician Empathy, JSPE
Results Pretest/post-test data on empathy scale showed no gender or age differences between the two groups. The mean total empathy score declined by 2.5 points during year 3 of medical school, the first full year of clinical experience. It is statistically significant (p<0.05).
***Hojat et al. (2005)^70^***
Aim To examine relationship between empathy, specialty, interest, personality and perceptions of mother and father.
Setting and Country Private medical school in Pennsylvania, USA
Design and Sample 422 medical students (1st year)
Rating Methods Jefferson Scale of Physician Empathy, JSPE & Zuckerman-Kuhlman Personality Questionnaire, ZKPQ
Results Women scored significantly higher on empathy than men (t(420)=4.58, p<0.01). Students who were interested in pursuing their future medical practice in people-oriented specialties obtained the highest empathy mean score and the differences were statistically significant (F(409.3)=7.93, p<0.01). The differences in empathy scores among the three groups with different levels of satisfaction with their mothers were statistically significant (F(417.2)=5.88, p<0.01). Significant correlation of law magnitude was found between empathy and sociability (r=0.15, p<0.01) and aggression-hostility (r=-0.13, p<0.01).
***Kataoka et al. (2009)^23^***
Aim To examine psychometric properties of a Japanese translation of the JSPE and to study differences in empathy scores between men and women and students in different years of medical school.
Setting and Country Okayama University Medical School, Japan
Design and Sample 400 medical students
Rating Methods Jefferson Scale of Physician Empathy, JSPE
Results Women outscored men by more than 3 points (107 and 103.7, respectively). The gender difference was statistically significant (t(376)=2.2, p=0.02). The mean empathy scores increased from 98.5 in the first year to 107.8 in the last year of medical school. The differences on mean scores in different years of medical school were statistically significant (F(5.394)=3.6, p=0.003).
***Magalhāes et al. (2011)^39^***
Aim The present cross-sectional analysis addresses the differences in empathy scores between the 1st year and senior students, between genders and between specialty preferences.
Setting and Country University of Minho, Portugal
Design and Sample 476 medical students
Rating Methods Jefferson Scale of Physician Empathy, JSPE
Results The measures for seniors (M=11.21, SD=9.10) were statistically higher than 1st year students (M=110.31, SD=10.63, F(1.387)=19.33, p<0.001). The empathy scores of female students (M=112.86, SD=10.81) were higher than the scores of male students (M=110.32, SD=10.69). No significant differences were found between students with a preference for people-oriented specialties (M=113.18, SD=10.92) and technology-oriented specialties (M=110.77, SD=10.52, F(1.387)=2.44).
***McKenna et al. (2011)^71^***
Aim To investigate empathy and attitudes towards specific medical conditions, two important aspects of the midwife–woman relationship amongst undergraduate midwifery students at one university,
Setting and Country Australia
Design and Sample 52 midwifery students
Rating Methods Jefferson Scale of Physician Empathy-Healthcare Provider, JSPE-HP & Medical Condition Regard Scale, MCRS
Results Overall reported a moderate degree of empathy (mean score=109.9, SD=20.9). An analysis of variance between the age groups showed no significant differences (P=0.112, p>0.05). Mean JSPE-HP empathy scores showed a steady increase from the 1st year (mean score=101.0, SD=28.5), to the 2nd year (mean score=110.35, SD=11.73) and to the final year (mean score=119.9, SD=12.6).
***Ozcan et al. (2010)^72^***
Aim To evaluate the empathetic skills and the empathetic tendency of nursing students throughout their years of undergraduate education.
Setting and Country Turkey
Design and Sample 438 nursing students
Rating Methods Empathetic Communication Skills Scale, ECSS & Empathetic Tendency Scale, ETS
Results There were no statistical differences among the year groups when comparing with the characteristics of the students (p>0.05). There were significant differences among the student groups (newly registered students, students who were at the end of 1st year, at the end of 2nd year, at the end of 3rd year and at the end of 4^th^ year) when comparing the ECSS and ETS scores (p<0.05). The ECSS scores of 4th year students were considerably higher than all groups. There was no difference between the ECSS scores of 2^nd^ and 3rd year students; however, the average scores of these both groups were higher than the scores of newly registered students were apparently higher than all of the other student groups(p<0.05). Students ECSS scores were more likely to increase, while ETS scores were more likely to decrease.
***Quince et al. (2011)^73^***
Aim Reports the findings in respect of two questions relating to university medical education: 1) Do men and women medical students differ in empathy? and 2) Does empathy change amongst men and women over time?
Setting and Country University of Cambridge, UK
Design and Sample Students from the 6 years of medical school
Rating Methods Interpersonal Reactivity Index, IRI
Results Women displayed statistically significant higher mean scores than men for affective empathy in all 6 years of medical training and cognitive empathy in 4 out of 6 years (years 1 & 2 and years 4 & 5). Amongst men, affected empathy declined slightly, although statistically significant, these changes were extremely small. Cognitive empathy was unchanged. Amongst women, neither affective nor cognitive empathy changed during the course.
***Roh et al. (2010)^74^***
Aim To evaluate the psychometric properties of the Korean version of the JSPE-S.
Setting and Country Seoul National University College of Medicine, South Korea
Design and Sample 493 medical students
Rating Methods Jefferson Scale of Physician Empathy-Student, JSPE-S
Results The mean scores of 5.1±0.7 and 5.2±0.6 were obtained for men and women, respectively, which did not significantly differ. In terms of school year, the mean scores were 5.1±0.6, 5.1±0.6, 5.0±0.6, 5.3±0.7 for 1st through 4th year of school, respectively, and the differences were statistically significant (p<0.05). Statistically significant difference for gender and school years, was found only among school years. The post hoc Tukey test showed the 4th year students to have significantly higher level of empathy than other cohorts (p<0.05).
***Shariat et al. (2010)^35^***
Aim To assess the psychometric properties of the Persian version of the JSPE in a sample of Iranian physicians and examine its correlates.
Setting and Country Iran
Design and Sample 207 general practitioners
Rating Methods Jefferson Scale of Physician Empathy, JSPE
Results Women had a higher mean empathy score than men (t=2.38, p=0.018) but marital status, place of practice, practice type or practice setting did not show any significant association with empathy. There was a significant positive correlation between empathy and both age and practice experiences (r=0.15, p=0.026; r=0.16, p=0.02).
***Shariat & Habibi (2013)^75^***
Aim To examine empathy in a large sample of Iranian medical students, and also to study factor structure and psychometric properties of the Persian translation of the JSPE-S.
Setting and Country 16 universities, Iran
Design and Sample 1187 medical students
Rating Methods Jefferson Scale of Physician Empathy-Student, JSPE-S
Results Mean scores for male and female students were 98.94 (SD=15.23) and 102.75 (SD=13.94), respectively. Female students scored significantly higher than males on the total score of JSPE (t(1160)=4.3, p<0.001). Comparison of students of 5 large universities with those of 12 smaller showed that the score of JSPE was significantly higher in large universities in preclinical students (t=2.17, p=0.03) and interns (t=2.22, p=0.03), but not in clinical trainees (t=-1.1, p=0.28). However, decreasing trend in empathy scores existed in both large and small universities from preclinical to clinical trainees and interns.
***Shashikumar et al. (2014)^76^***
Aim There is a need to understand empathy and its correlates among medical students in India.
Setting and Country Armed Forces Medical College, India
Design and Sample 488 medical students
Rating Methods Jefferson Scale of Physician Empathy-Student, JSPE-S
Results No difference in empathy across various semesters of those who chose people-oriented and technology-oriented specialties, but the undecided students had significant increase (df=4, p=0.0002). There is a decline in empathy after first semester, but especially more in seventh semester. Significant decline in empathy was evident with time spent in undergraduate medical education. Female students had no significant decline in empathy across various semesters and male students showed a significant decline in third and seventh semesters.
***Suh et al. (2012)^26^***
Aim To evaluate the psychometric properties of a Korean version of the JSPE among Korean physicians.
Setting and Country South Korea
Design and Sample 229 physicians
Rating Methods Jefferson Scale of Physician Empathy, JSPE
Results Women scored higher than men by 3.8 points and the difference was statistically significant (t(227)=2.35, p<0.05). Mean scores for physicians practicing dermatology, internal medicine and rehabilitation medicine were significantly higher than those in general practice, radiology and other specialties (F(7.221)=3.84, p<0.01).
***Ward et al. (2012)^77^***
Aim To examine changes in empathy during an academic year among undergraduate nursing students.
Setting and Country Thomas Jefferson University, Jefferson School of Nursing, USA
Design and Sample 214 undergraduate nursing students
Rating Methods Jefferson Scale of Empathy, JSE
Results There was a decline in empathy in the total sample, which was statistically significant (t(212)=1.97 p=0.05) but it was not practically important as indicated by the effect size of -0.16. The decline was statistically and practically important for Asian oriented students (d=-0.62) and for students with undergraduate degrees in business (d=-1.37) and in science (d=-0.46). Significant decline in empathy was observed among students with varied patient exposure and clinical experiences during nursing school (F(2.211)=4.2 p<0.1). The decline was practically important for students with more clinical exposure than for those with limited clinical experience. Prior work experience in clinical settings were associated with a significant decline in empathy. There was not found significant relationship between student ages and scores on the JSE (r=0.12 for pretest and posttest scores r=0.06).
***Wen (2013)^78^***
Aim To examine the psychometric properties of JSPE-S among a sample of Chinese medical students.
Setting and Country China
Design and Sample 902 clinical medical students
Rating Methods Jefferson Scale of Physician Empathy-Student, JSPE-S
Results Statistically significant difference between genders. The difference between mean scores from various years of medical school were statistically significant (F=3.08, p<0.05). The mean empathy score of 1st year and 4th year students had a significant difference (p=0.034 p<0.05) The differences between age groups (t=1.29 p=0.20) were not statistically significant.
***Williams et al. (2014)^79^***
Aim To examine the extent and nature of empathy among emergency health, nursing and midwifery students at one Australian university and to investigate the longitudinal changes in empathy levels across the course of the study.
Setting and Country Monash University, Australia
Design and Sample 948 emergency health, nursing and midwifery students in the 1st, 2nd and 3rd year.
Rating Methods Jefferson Scale of Physician Empathy-Healthcare Provider, JSPE-HP
Results Midwifery students’ mean score (M=108.98, SD=17.2) was higher than those for emergency health (M=104.41, SD=14.9) and nursing students (M=103.92, SD=14.4). There was a statistically significant difference at the p<0.05 level in empathy scores between the 3 undergraduate courses F(2.945)=7.74, p<0.0001. The mean score for midwifery students was significantly different at the p<0.05 level. Women’s mean score (M=106.2, SD=14.83) was higher than men’s (M=100.6, SD=14.41) but it was not statistically significant (t(1936)=2.05, p=0.635). Compared by age students in the age range of 26–30 years and 31–35 years recorded higher empathy scores than their younger colleagues aged <21 years and 21–25 years and the difference in mean scores was found to be statistically significant at the p<0.05 level, F(6.939)=3.83, p<0.001. When compared by year level, students in the 2nd (M=106.50, SD=13.03) and 3rd (M=104, SD=16.34) years reported higher empathy scores than those in the 1st year (M=103.82, SD=16.80) but the difference was not statistically important.

In another study, empathy appears to increase during the 1st year of medical school but decreases after the 3rd year (1st clinical year) and remains low through the final year of medical school^65^. The results are consistent with previous studies suggesting that empathy decreases after clinical training in medical school (Table1).

A study hypothesised that empathy would increase with time in medical school. The latent growth model (LGM), refuted the hypothesis showing that JSPE scores were longitudinally stable. There is a gender-related evolution that globally results in a linear non-significant growth that is not hampered by the preclinical/clinical transition. A negative change in empathy was hypothesised at the transition from the preclinical to clinical training, but the data did not confirm it, as the empathy levels grew from the entrance in the medical degree to the start of the clinical training. However, a significant decline in empathy was found in female students in the transition period. As far as it concerns personality, students with higher scores on openness and agreeableness subscales scored higher in JSPE upon admission to medical school. Overall this study disagrees with the notion that empathy declines throughout medical training^66^ (Table 1).

A multi-institutional and cross-sectional study in Portugal^67^ suggested that medical students who were more agreeable and open to experience were also likely more empathetic. This conclusion reinforces the argument that the personality and empathy of medical students are related (Table 1).

An Italian study expected a significant gender difference in empathy scores in favour of women. Although a slightly higher score was observed for women, the difference was not statistically significant. This unexpected finding probably could be due to a volunteer-sample factor. Only a slight difference was found in empathy scores between medical and surgical specialities, but it was not statistically significant. It is interesting to note that there were statistically significant differences observed in the scores of physicians from different hospitals. The hospital where the physicians scored higher was the only one devoted to the diagnosis and therapy of a specific patient population. Therefore, patient–physician relationships may last longer in such a hospital, which can contribute to the relatively higher empathy scores of physicians there^6^ (Table 1).

A study that took place in Kuwait found that students whose mothers had not completed high school had lower levels of empathy compared to students whose mothers were more educated. The mean empathy score among medical students in Kuwait University on the JSPE is relatively lower than that of medical students in Western countries. However, the mean empathy score is comparable to that reported from Asian countries. The higher level of empathy found among female students is consistent with other studies. The relationship between empathy scores and monthly household income was found to be significant; students coming from families with lower incomes were significantly less empathetic. This may be due to the role of the educational status of the parents and the family income, which may affect the way students develop certain emotional skills and hence empathy. As empathy is an individual characteristic that is shaped by interpersonal relationships, people who have enjoyed positive relationships with their mothers throughout their upbringing are more likely to display empathy in social situations. No statistically significant associations between personality traits and empathy scores were found. Furthermore, a weaker positive correlation between stress levels and empathy scores was found, indicating that those with higher stress are more empathetic than those with low-stress scores^68^ (Table 1).

Hojat and colleagues who have created the Jefferson Scale of Physician Empathy have conducted many studies on empathy. In 2002, a study in Thomas Jefferson Hospital showed that women tend to score higher on empathy ratings than men, although in this case, the finding did not reach statistical significance. But it falls short of explaining gender differences in empathy. The significant differences in empathy scores observed among physicians in various specialities might reflect the notion that different individuals with different degrees of interpersonal skills, reflected in their empathy scores, are attracted to different specialities^22^. The result of the 2004 study is consistent with findings of other studies that observed a decrease in emotional empathy before and following clinical experiences among medical students^69^. In the 2005 study, the Zuckerman-Kuhlman Personality Questionnaire (ZKPQ) was also used and the findings generally suggested that empathy among medical students is a function of gender and early relations with the mother^70^ (Table 1).

In a Japanese study, the mean score for the Japanese sample was lower than that reported in Western countries, and it is possible that differences between Japanese and Western cultures might be playing a role. Although the finding that female medical students score higher than their male counterparts is also present here. The finding regarding enhancement of empathy during medical school in Japanese students is not in agreement with that reported for American medical students, where there is a decrease in empathy. The different curricula could be responsible for this difference^23^ (Table 1).

The findings of the Portuguese study^39^ are similar to those of past studies undertaken with 6-year undergraduate medical programs with Japanese and Korean versions of the JSPE. It also identifies differences in JSPE scores by gender, thus confirming findings from previous reports (Table 1).

The mean empathy score of an Australian cohort^71^ of undergraduate midwifery students is somewhat lower than the mean empathy score reported in other studies using the JSPE-HP. There was no significant difference between the age groups of midwifery students in the study.

The findings reported by a Turkish study showed that ECSS scores of 4th year nursing students were significantly higher than all other student groups. However, ETS scores of newly registered students were higher than other groups. Further, the longitudinal assessment also showed that ECSS scores became higher, but ETS scores became lower during the undergraduate years (p<0.05). The findings show that during the nursing education years, students may learn how to respond to others’ feelings and needs. However, according to the current study, there was no correlation between ETS and ECSS scores^72^ (Table 1).

In medical schools in the UK, the first three years are the core science component and the last three years the clinical component. In a study that took place in the UK^73^, it was observed that there were statistically significant gender differences in affective empathy in all six years and in cognitive empathy for four of the six years. Differences in mean scores between men and women were larger than any of the changes in mean scores between the stages of the course. It was found that with time, affective empathy declined on average for men, while sensitivity analysis revealed that women’s affective empathy declined during the clinical component. Amongst women during the core science component, affective empathy remained constant, on average. There were no significant changes in cognitive empathy among women or amongst clinical men. Sensitivity analysis revealed that during the core science component of the course men’s cognitive empathy increased. However, although these changes were statistically significant, regression coefficients indicate that they were minimal and therefore of questionable practical significance (Table 1).

Another study revealed that Korean medical students’ empathy scores were lower than those of their US counterparts, similar to the Japanese findings. The finding that female students have higher scores than male students is consistent with previous studies, although the difference was not statistically significant. Slightly lower empathy scores in 3rd year students were observed than in other years, though the difference was not statistically significant. Unexpectedly, there was a small but significant increase in empathy in both 4th year male and female students^74^ (Table1).

In a 2010 study of physicians’ empathy in Iran, the scores in females were higher than in males, consistent with most other studies^35^. A significant association between age and empathy, after controlling for practical experience, was not found in this study. Also consistent with other Eastern studies was the lower mean empathy score, lower than the Western studies but higher than in Japan. In the study of medical students’ empathy in Iran^75^, female students scored significantly higher than male students, again consistent with previous studies. There was a decreasing trend in empathy score from preclinical to clinical trainees and interns, for large and small universities. However, this occurred at a different point in time in large universities versus small universities. In large universities, the erosion of empathy took place in the transition from preclinical to clinical training, whereas in the small universities, it happened after some years of clinical training and the beginning of the internship period (Table 1).

A study conducted in 2014 in India^76^ showed that empathy declined during medical education, but it reached a significant level only in the seventh semester. It also showed that female students have significantly higher empathy than male students. However, empathy level increased significantly (p=0.0002) from first to ninth semester among those choosing specialities other than people/technology-oriented specialisations or remaining undecided. A decline in the score was noticed in the third semester (beginning of the second year), but it was statistically not significant. Interestingly, there was an increase in empathy scores in the fifth semester (beginning of the third year). Like in other studies there was a significant difference (p=0.012) in empathy scores between male and female students, with the females scoring more than males (mean female students 106.5 vs male students 101.89, standard deviation 19.901 and 16.164, respectively). A significant difference (mean=11.103) is seen among male students from first to seventh semester (p=0.020). Thus, there is a need to probably focus on improving male students’ empathy, especially those of seventh semester (entering the fourth year). Female students were, by their nature, more caring and loving, and probably less affected by factors that tend to diminish empathy. The undecided students about speciality or those choosing other subjects had the highest mean empathy while those choosing people-oriented subjects had the least empathy score (Table 1).

Except for the study in Korean medical students^74^, another study in Korean physicians was conducted in 2012^26^ and revealed that the mean empathy score for Korean physicians (mean=98.2, SD=12.0) was lower than that reported for Korean medical students (mean=103.1, SD=12.5). The score was also lower than that reported for American (mean=120, SD=12) and Italian (mean=115.1, SD=15.55) physicians. The physicians in the so-called ‘people-oriented’ specialities tend to have higher empathy scores than their counterparts in the ‘procedure-oriented’ specialities such as radiology. Lastly, the empathy score for women was significantly higher than that for men (Table 1).

Research that studied the empathy scores for nursing students showed a significant decline in mean empathy scores for particular groups of undergraduate nursing students. The participants were from three undergraduate nursing programs: a) Associate degree (ADN, n=120, Group 1) first and second-year students; b) Bachelor degree (BSN, n=60, Group 2) third and fourth-year students; and c) Facilitated Academic Coursework Tract students (FACT, n=34, Group 3). Students admitted to the FACT have earned a previous degree from another discipline. The magnitude of the decline in empathy was similar for nursing students in Groups 2 and 3, who were more exposed to patient encounters than their counterparts in Group 1. The findings of this study are consistent with several research studies that have found similar differences in the decline of empathy among medical students, particularly those studies that reported a significant erosion of empathy in the third year of medical school when students start their formal clinical training with real patients^77^ (Table 1).

A study in Chinese medical students^78^ showed again that empathy in Eastern countries is lower than Western countries, since the Chinese mean empathy score (mean=109.60, SD=13.34) was lower than that for American students (mean=115, SD=10), but higher than that for Japanese students (mean=104.3, SD=13.1) and Iranian students (mean=105.1, SD=12.9). This may be attributed to cross-cultural differences in social norms, ethnicity, religious beliefs, pedagogical methods, and sex stereotyping, which can influence empathetic engagement. The empathy scores in the 1st year are the lowest. The demographic characteristics of the younger students suggest a relative like for life experience and may result in the initial lower levels of empathy. A significant difference in empathy scores between different genders was found, which was in favour of women (p<0.05, t(753)=5.57), consistent with most other studies. The gender difference in empathy has been attributed to intrinsic factors (e.g. evolutionary-biological gender characteristics) as well as extrinsic factors (e.g. styles in interpersonal care, socialisation and gender-role expectations). This explanation has been widely accepted and supported in the relevant literature. There were statistical differences in empathy scores among medical students in different years of medical school (F=3.08, p<0.05) with the 1st year students having the lowest empathy scores (mean=107.36) and the 4th year medical students having the highest (mean=112.12) (Table 1).

Studies in other health science students except medicine and nursing are not frequent. There was a study in an Australian university that along with the empathy of nursing students they also measured the empathy of emergency health and midwifery students. The findings demonstrate that female students had a higher mean JSPE-HP empathy score compared with male students, although it did not reach statistical significance. Mean empathy scores for this cohort of undergraduate health science students are lower than the scores reported in other studies that used the JSPE-HP. The reporting of no statistical difference between each of the three years across the health professional courses in this study is an important finding as it suggests the courses do not have a detrimental effect on student empathy^79^ (Table 1).

## DISCUSSION

During the review, it was observed that in most studies women had higher levels of empathy than men, in both students and health professionals^65,6,68,22,70,23,39,73,35,75,26,78,79^. In three studies only, there were no differences between the levels of empathy based on gender^64,69,74^. Age does not seem to affect empathy, with only two studies finding a positive correlation^35,79^. Concerning the progress of empathy in students, there is no agreement, with several studies suggesting that empathy increases in the last year of education^64,68,23,39,71,72,74,78,79^ and others reporting that with the beginning of clinical experience the empathy declines^65,69,75,77^. In one study only the empathy levels of women declined from preclinical to clinical stages^67^. Students and health professionals that had chosen a people-oriented speciality had higher levels of empathy than those who chose a technology-oriented specialization^65,6,22,70,26^. In two studies there were no differences^39,76^. There is a belief that empathy is a personality trait and as such, it can be influenced by the other traits of personality. A positive correlation was found between empathy and ‘Openness to Experience’, ‘Agreeableness’, and ‘Conscientiousness’^66,67,70^. A negative correlation was found between ‘Aggression-Hostility’ and empathy^70^. Also, according to one study, higher stress levels were linked to higher levels of empathy^68^. Furthermore, a good relationship with the mother suggested higher levels of empathy^68,70^. However, although empathy is so important in midwifery care, the number of research studies of the level of empathy in midwives is extremely limited. For this reason, these types of studies are warranted and need to be expanded. Even though there are few studies measuring midwives’ empathy, they show that midwives have high levels of empathy and empathy among midwifery students rises during the years of education. Specifically, in an Australian study^71^, the undergraduate midwifery students had a somewhat lower mean empathy score. The difference was not significant, but such a positive gradation in mean empathy is a very positive result as it indicates that the course has a positive effect on students’ professional development. One study shows that midwifery students’ mean score (M=108.98, SD=17.2) was higher than those for emergency health (M=104.41, SD=14.9) and nursing students (M=103.92, SD=14.4)^79^. A recent study found that when midwives’ empathy and spiritual care were evident, women’s birth experiences appeared positively enhanced, providing a solid foundation for confident mothering. In the same study, participants appeared to link a lack of caregiver empathy, compassion or spiritual care with more enduring negative consequences, such as birth trauma and difficulty bonding with their babies^80^.

However, although empathy is so important in midwifery care, the number of researchers who study the level of empathy in midwives is extremely limited. For this reason, there is a need to expand these kinds of studies. Recently, in 2016, a scale that measures midwives’ empathy was developed (Midwifery Empathy Scale - MES), which is very promising and opens the way for further research in midwifery^51^.

### Limitations

This review has some limitations. The review included papers published in English, and for this reason, important studies may have been overlooked. Also, we did not have access to some studies because a subscription was required.

### Recommendations for future research

It is widely accepted in the literature that empathy is a multidimensional concept and there is no agreement on its definition. As a result, many questions may arise, not only for its definition but also for its components and that is why there is a need for further research. There is disagreement on whether empathy is a personality trait or a skill that can be taught and improved, and on the factors that contribute to its development. Some studies are suggesting that parent–child relationships and family environment affect empathy, but further exploration is required. The cultural factor may also be relevant. Furthermore, could educational programs and studies of literature and the arts enhance empathy?

## CONCLUSIONS

Studies have begun to investigate the neurobiological aspect of empathy, but more research is needed to understand how empathy works fully; the same applies to studies assessing the relations between personality traits and empathy. There is a necessity to investigate the long-term effects of health professional empathy on patient satisfaction, clinical outcome and malpractice litigation. Most of the existing instruments for measuring empathy are self-rating. A validated instrument that measures empathy of health professionals as viewed from the patients’ perspective is required to investigate whether the overlap is found between self-reporting and patient-reporting scores of empathy. In our review, the Jefferson Scale of Physician Empathy is the most frequently used scale, but there are many existing scales and a systematic review examining all of them would be useful. Furthermore, empathy is a very helpful and a worthwhile trait of a midwife, and for this reason, there is a great necessity for new studies. Midwifery Empathy Scale (MES) is a new scale measuring midwives’ empathy and is an important tool that will help to expand these studies.
